# *Macrosemiafengi* Wang sp. nov. from Yunnan and Guizhou, China (Hemiptera, Cicadidae, Cicadinae)

**DOI:** 10.3897/BDJ.12.e115974

**Published:** 2024-01-31

**Authors:** Cheng-Bin Wang

**Affiliations:** 1 Engineering Research Center for Forest and Grassland Disaster Prevention and Reduction, Mianyang Normal University, 166 Mianxing West Road, Mianyang, China Engineering Research Center for Forest and Grassland Disaster Prevention and Reduction, Mianyang Normal University, 166 Mianxing West Road Mianyang China

**Keywords:** cicada, Dundubiini, taxonomy, new species, morphology, distribution, Oriental Region

## Abstract

**Background:**

The genus *Macrosemia* Kato, 1925 (Hemiptera, Cicadidae, Cicadinae, Dundubiini, Dundubiina) currently includes 16 species (excluding subspecies and varieties), mainly occurring in the Oriental Region. More than half of them, 10 species, are known from China, including one new species, described in the present study.

**New information:**

A new species of cicada, *Macrosemiafengi* Wang **sp. nov.**, is described from Yunnan and Guizhou, southwest China. Colour plates are presented to illustrate its diagnostic characters. The distribution map of the new species is also given.

## Introduction

In the 20^th^ century, all species belong to *Macrosemia* Kato, 1925 (Hemiptera, Cicadidae, Cicadinae, Dundubiini, Dundubiina) were described before 1940. After more than sixty years, in this century, the taxonomic study on *Macrosemia* is flourishing again. Three species were successively described by Boulard (2001), Boulard (2003) and Boulard (2008): *M.khuanae* Boulard, 2001 and *M.beaudouini* (Boulard 2003) (transferred by Lee (2014) from *Orientopsaltria* Kato, 1944) both from Thailand and *M.suavicolor* Boulard 2008 from Vietnam. Lee (2008) transferred five species from *Platylomia* Stål, 1870 to *Macrosemia*: *P.assamensis* Distant, 1905, *P.diana* Distant, 1905, *P.divergens* (Distant, 1917), *P.pieli* Kato, 1938 and *P.saturata* (Walker, 1858). In the most recent and influential catalogue on cicadas worldwide, Sanborn (2013) catalogued 14 species in *Macrosemia*, with 10 of them being known from China. Soon after, Wang and Wei (2014) and Lee (2014) coincidentally transferred *Platylomiajuno* Distant, 1905 to *Macrosemia* in the same year. Most recently, two species, *M.lamdongensis* Pham, Bui & Constant, 2016 and *M.sapaensis* Luu, Pham, Bui & Constant, 2022, were described from Vietnam (Pham et al. 2016 and Luu et al. 2022). In addition, Hayashi and Usui (2017) put *M.diana* (Distant, 1905) and *M.pieli* Kato, 1938 back to the original combination *Platylomia*.

*Macrosemia* is distinguishable from its allied genera, especially from *Platylomia*, by the following characteristics (modified from Chou et al. (1997), Lee and Hayashi (2003), Lee (2008), Wang and Wei (2014), Pham et al. (2016), Luu et al. (2022) and Luu et al. (2022)): 1) body large, robust and thick; 2) eyes not prominent laterally; 3) head about as wide as or narrower than mesonotal base; 4) postclypeus a little swollen anteriorly, shorter than median length of vertex in dorsal view; 5) ventral side of postclypeus, in many species, with distinct fuscous or black “Y”-like fascia; 6) pronotal collar well developed, broad and long, about 0.4 times as long as median length of pronotum disc; 7) mediolateral tooth of pronotal collar produced laterally or posterolaterally, not anterolaterally; 8) mesonotum excluding cruciform elevation distinctly longer than pronotum; 9) male abdomen nearly as long as, not distinctly longer than distance from head to cruciform elevation; 10) tymbal concealed by timbal cover in dorsal view, but a little exposed laterally; 11) male operculum long with comparatively narrow apex and swollen at about posterior two-thirds; 12) forewing hyaline, slender with narrow tip, longer than 3.2 times of width; 13) ovipositor sheath extending a little beyond dorsal beak.

Here, I describe and illustrate a new species under the name of *Macrosemiafengi* **sp. nov.**, which is in accordance with all the above characteristics except 5, from Yunnan and Guizhou Provinces of China. Important morphological characteristics of the new species are illustrated and the known distribution is mapped.

### Species list of Macrosemia Kato, 1925 from China

(Alphabetically listed and modified from Sanborn (2013))

1. *Macrosemiaanhweiensis* Ouchi, 1938 (Anhui);
2. *Macrosemiaassamensis* (Distant, 1905) (= *Platylomiaassamensis*) (Yunnan; India, Thailand);
3. *Macrosemiadivergens* (Distant, 1917) (= *Cosmopsaltriadivergens* = *Platylomiadivergens* = *Orientopsaltriadivergens*) (Yunnan; Laos, Thailand);
4. *Macrosemiafengi* Wang, **sp. nov.** (Guizhou, Yunnan);
5. *Macrosemiakareisana* (Matsumura, 1907) (= *Cosmopsaltriakareisana* = *Platylomiakaraisana* (sic) = *Platylomiakareisana* = *Platylomiakareipana* (sic) = *Marosemiakaraisana* (sic) = *Platylomiahopponis* Kato, 1925 = *Macrosemiahopponis* = *Macrosemiakareisanahopponis* = *Platylomiakarapinensis* Kato, 1925 = *Macrosemiakareisanakarapinensis* = *Cosmopsaltriamontana* Kato, 1927 = *Orientopsaltriamontana*) (Taiwan; Japan);
6. *Macrosemiakiangsuensis* Kato, 1938 (= *Platylomiakingvosana* Liu, 1940 = *Platylomiakinvosana* (sic)) (Jiangsu);
7. *Macrosemiajuno* (Distant, 1905) (= *Platylomiajuno* = *Cosmopsaltriajuno*) (Hainan, Sichuan, Xizang; Laos);
8. *Macrosemiamatsumurai* (Kato, 1928) (= *Macrosemiamatsumura* (sic) = *Macrosemiamatsumarai* (sic) = *Platylomiamatsumurai*) (Fujian, Hunan, Taiwan; Japan);
9. *Macrosemiatonkiniana* (Jacobi, 1905) (= *Cosmopsaltriatonkiniana* = *Cosmoscarta* (sic) *tonkiniana* = *Platylomiatonkiniana* = *Orientopsaltriatonkiniana*) (Hainan, Yunnan; Laos, India, Myanmar, Thailand, Vietnam);
10. *Macrosemiaumbrata* (Distant, 1888) (= *Cosmopsaltriaumbrata* = *Platylomiaumbrata* = *Macrosemiachantrainei* Boulard, 2003) (Yunnan; Bhutan, Laos, India, Myanmar, Nepal, Sri Lanka, Thailand).

## Materials and methods

Two males and one female of the new species were collected from Yunnan of China in September 2022 and a month later, two pairs of the same species were collected from Guizhou of China. After field works, the specimens were kept in a freezer (-20℃). About half a year later, the specimens were relaxed and softened in water at room temperature for 24 hours and then placed in distilled water for cleaning and dissection. To examine the male genitalia, the pygofer (containing the aedeagus), together with sternite VIII, were detached and treated with a 10% potassium hydroxide (KOH) solution at room temperature for 12 hours. They were then placed in distilled water to remove the remaining KOH and prevent any further bleaching. After examination, the body parts were mounted on a slide using Euparal Mounting Medium for future studies. Images were taken with a Canon macro photo lens MP-E 65 mm on a Canon 5DsR. Images of the same object at different focal planes were combined using Zerene Stacker 1.04 stacking software. Adobe Photoshop CS6 was used for postprocessing. The description was carried out on dry specimens. Morphological terminology follows Moulds (2005) and Moulds (2012) and higher taxonomy follows Marshall et al. (2018) and Simon et al. (2019). Measurement criteria in millimetres (mm) follows Wang and Liu (2022).

The type material of the new species is deposited in the following institutional and private collections:

- **MYNU** - Invertebrate collection of Mianyang Normal University, Mianyang, China;
- **cLFW** - private collection of Lei Feng, Weifang, China.

## Taxon treatments

### Macrosemia fengi

Wang sp. nov.

FC7012CB-2BBC-5AC8-93C5-860207232C63

02E5D426-AEC8-4B76-AD7D-CEDF26D48FA3

#### Materials

- **Type status:** Holotype. **Occurrence:** recordedBy: Yun-He Wang; sex: male; occurrenceID: AAC314B2-C4AE-5770-8270-AB699809FF57; **Location:** country: China; stateProvince: Yunnan; verbatimLocality: Xishuangbanna Prefecture, Menghai County, Mengsong Township, Da’an Village [大安村]; **Event:** verbatimEventDate: 22.IX.2022; **Record Level:** collectionID: WCBA00170; institutionCode: MYNU
- **Type status:** Paratype. **Occurrence:** recordedBy: Yun-He Wang; sex: 1 male; occurrenceID: 68B72A41-13B7-517F-A16A-1422815F564A; **Location:** country: China; stateProvince: Yunnan; verbatimLocality: Xishuangbanna Prefecture, Menghai County, Mengsong Township, Da’an Village [大安村]; **Event:** verbatimEventDate: 22.IX.2022; **Record Level:** collectionID: WCBA00171; collectionCode: cLFW
- **Type status:** Paratype. **Occurrence:** recordedBy: Yun-He Wang; sex: 1 female; occurrenceID: D19FC1B1-64C6-585A-9858-102421689510; **Location:** country: China; stateProvince: Yunnan; verbatimLocality: Xishuangbanna Prefecture, Menghai County, Mengsong Township, Da’an Village [大安村]; **Event:** verbatimEventDate: 22.IX.2022; **Record Level:** collectionID: WCBA00172; collectionCode: cLFW
- **Type status:** Paratype. **Occurrence:** recordedBy: Wei Li; sex: 1 male; occurrenceID: 6D7CB34C-CB21-5C4D-8F54-6D0DDC8EB153; **Location:** country: China; stateProvince: Guizhou; verbatimLocality: Guiyang City, Wudang District, Xinbao Township, Xiangzhigou Scenic Area [香纸沟风景区]; **Event:** verbatimEventDate: 22.X.2022; **Record Level:** collectionID: WCBA00173; institutionCode: MYNU
- **Type status:** Paratype. **Occurrence:** recordedBy: Wei Li; sex: 1 female; occurrenceID: 54A6D4CE-C8F7-5556-BCBA-410DEBCADF16; **Location:** country: China; stateProvince: Guizhou; verbatimLocality: Guiyang City, Wudang District, Xinbao Township, Xiangzhigou Scenic Area [香纸沟风景区]; **Event:** verbatimEventDate: 22.X.2022; **Record Level:** collectionID: WCBA00174; institutionCode: MYNU
- **Type status:** Paratype. **Occurrence:** recordedBy: Wei Li; sex: 1 male; occurrenceID: 4B5F3DEA-6481-510A-9BF6-3099B51F3863; **Location:** country: China; stateProvince: Guizhou; verbatimLocality: Guiyang City, Wudang District, Xinbao Township, Xiangzhigou Scenic Area [香纸沟风景区]; **Event:** verbatimEventDate: 22.X.2022; **Record Level:** collectionID: WCBA00175; collectionCode: cLFW
- **Type status:** Paratype. **Occurrence:** recordedBy: Wei Li; sex: 1 female; occurrenceID: 6494093A-7E5F-56B0-9098-03415307F758; **Location:** country: China; stateProvince: Guizhou; verbatimLocality: Guiyang City, Wudang District, Xinbao Township, Xiangzhigou Scenic Area [香纸沟风景区]; **Event:** verbatimEventDate: 22.X.2022; **Record Level:** collectionID: WCBA00176; collectionCode: cLFW

#### Description

**Figure 1.**
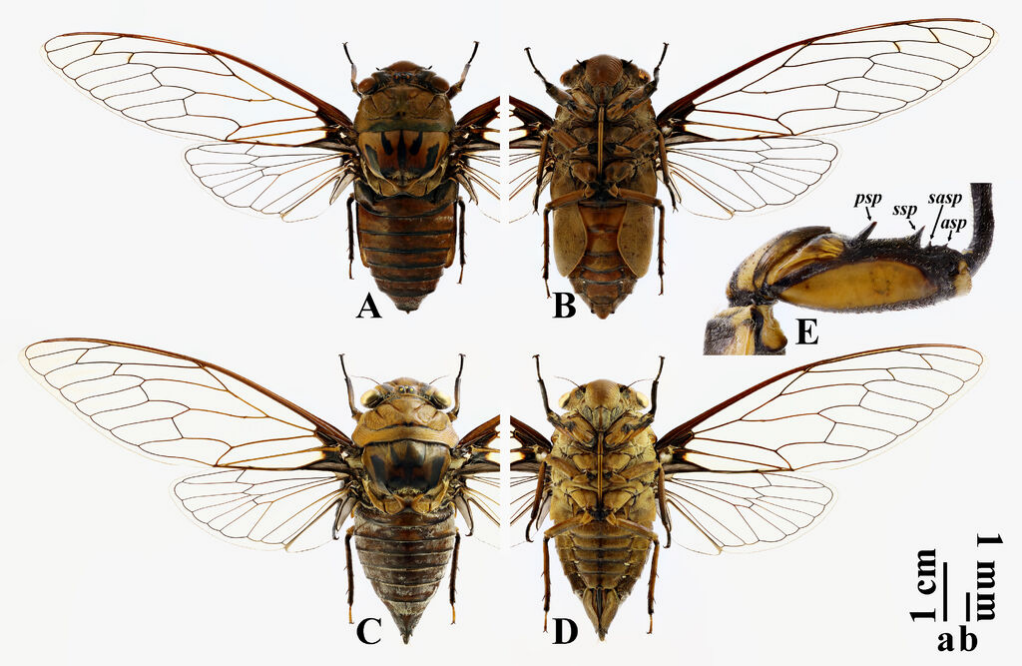
*Macrosemiafengi* Wang **sp. nov.**: **A, B** habitus, holotype, ♂; **C, D** habitus, paratype, ♀; **E** fore femur, holotype, ♂. **A, C** dorsal views; **B, D** ventral views; **E** lateral view. Abbreviations: asp: apical spine; psp: primary spine; sasp: subapical spine; ssp: secondary spine. Scale bar a for A–D; b for E.

**Male** (Fig. 1A and B). Measurements (n = 4) as shown in Table 1. Ratios of different body parts: (pronotal length)/(head length) = 1.9; (mesonotal length excluding cruciform elevation)/(pronotal length) = 1.2; (abdominal length)/(head + pronotal + mesonotal length) = 0.9; (head width)/(pronotal width) = 0.9; (head width)/(mesonotal width) = 1.0; (abdominal tergite III width)/(mesonotal width) = 1.1; (forewing length)/(forewing width) = 3.4.

Head with bottom colour brown, with following blackish markings: median fascia inverted “U”-like, enclosing three ocelli, reaching frontoclypeal suture anteriorly and basal margin of head posteriorly; two fasciae on each supra-antennal plate, one narrow and along medial margin, another one relatively wide and obliquely in posterior part; lateral fasciae large, between median fascia and eyes. Compound eyes brown. Ocelli reddish-brown. Distance between lateral ocellus and corresponding eye about 2.5 times as wide as distance between lateral ocelli. Antennae fuscous to blackish. Postclypeus a little swollen, entirely brown. Anteclypeus brown in median part and blackish laterally. Genae blackish in anterior part and brown posteriorly. Lorum almost entirely blackish. Rostrum brownish, except blackish at apex, just reaching metacoxae.

Thorax. Pronotum almost entirely brown at both pronotal disc and pronotal collar, faintly tinged fuscous in central part of pronotal disc and posterior part of pronotal collar, without markings along paramedian and lateral fissures, but particularly with paired blackish submedian spots near ambient fissure at pronotal disc. Pronotal collar with median length relatively long, about 0.4 times as long as that of pronotum disc, slightly ampliate posterolaterally; lateral margins with roundly obtuse lateral teeth at about anterior one-third, orientating posterolaterally; hind corners widely rounded; surface transversely grooved. Mesonotum brownish, tinged reddish in central part, with following blackish markings: median fascia long and slender, broadened in middle part, with apex bifurcated and reaching anterior margin of cruciform elevation; paramedian fasciae short, broadened posteriorly, along parapsidal sutures; lateral fasciae wide, “L”-like, starting from about anterior one-fifth of mesonotum, somewhat obliquely extending to anterior arms of cruciform elevation, then turned medially to enclose scutal depressions, not confluent with median fascia; marginal fasciae small and short, hardly seen from dorsal view, along posterior parts of mesonotal lateral margins. Cruciform elevation brownish, fuscous to blackish in posterior arms and apical parts of anterior arms. Wing grooves brownish. Thoracic sternites almost all brownish.

Legs bicoloured, with bottom colour brown; profemora with blackish oblique stripes; meso- and metafemora with two or three blackish spots at apices. Profemur (Fig. 1E) with four spines: primary spine long, subdigitiform, with obtuse apex; secondary spine subtrianglular and rather sharp; subapical spine small and tuberculate; apical spine rather small and sharp. Meracanthi mostly brown with blackish margins.

Wings hyaline. Venation generally brownish in basal part and fuscous apically; R+Sc veins reddish fuscous. Forewing with 8 apical cells; ulnar cell 3 about 1.5 times as long as apical cell 5; RA_2_ vein with longitudinal portion about 2.9 times as long as basal portion; infuscations present on r and r-m crossveins; nodal line absent; basal cell blackish; basal membrane greyish-brown. Hind-wing with 6 apical cells; jugum and longitudinal margins of vannus greyish-brown.

**Figure 2.**
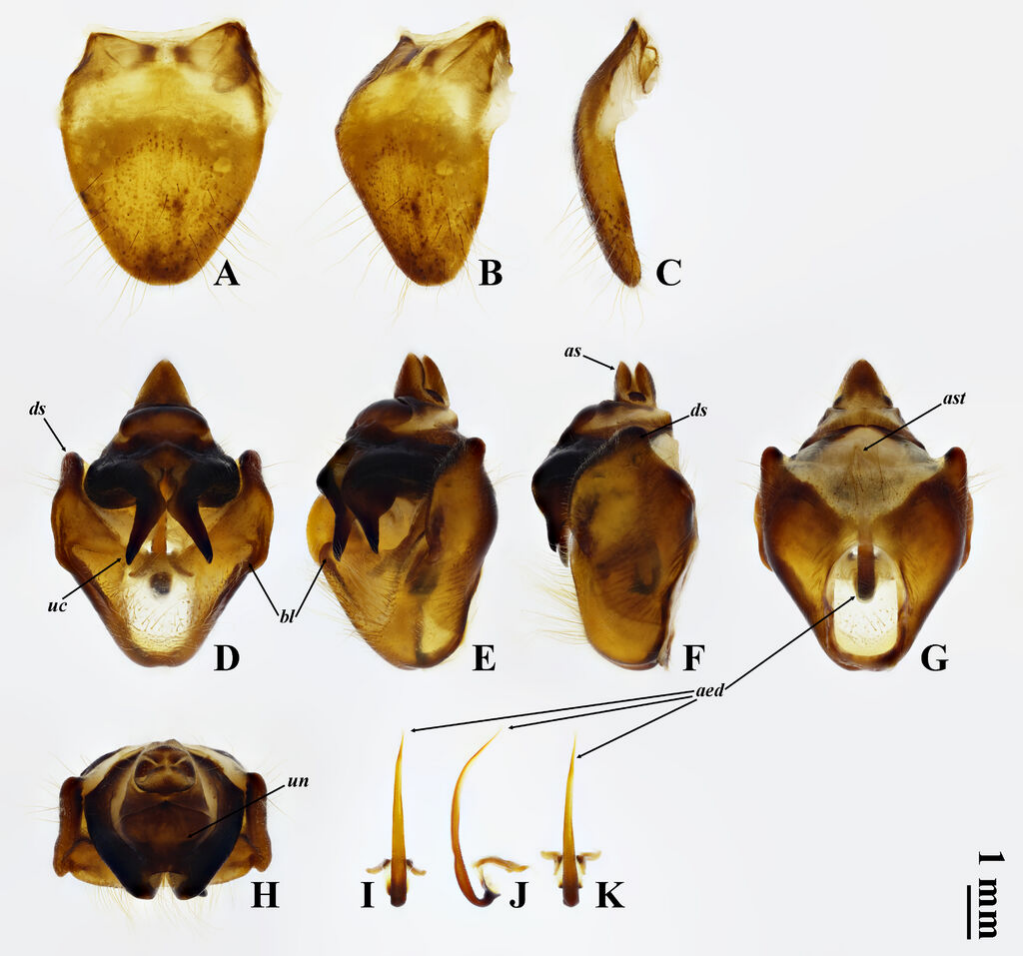
*Macrosemiafengi* Wang **sp. nov.**, holotype, ♂: **A–C** abdominal sternite VIII; **D–H** pygofer; **I–K** aedeagus. **A, D** ventral views; **B, E** ventrolateral views; **C, F, J** lateral views; **G, I** dorsal views; **H, K** apical views. Abbreviations: aed: aedeagus; as: anal styles; ast: apical stylus; bl: basal lobe; ds: distal shoulder; uc: uncal clasper; un: uncus.

Abdomen subcylindrical, gradually narrowing posteriorly; reddish fuscous on dorsum and brown on venter, with posterior margin of each tergite narrowly blackish, without distinct white pollinosity. Timbal cover scalelike, brown in lateral half and blackish in medial half, concealing timbal in dorsal view. Operculum entirely brownish, except blackish lateral margin; elongate, with margins moderately constricted in sub-basal part; apex rounded, extending to middle level of abdominal sternite VII; separated from each other a little narrower than one width of them. Abdominal tergite III 1.1 times as wide as mesonotum. Abdominal sternite VII wider than long, slightly emarginate in middle of posterior margin; sternite VIII (Fig. 2A–C) subcordiform, gradually narrowing posteriorly, with posterior margin rounded; surface distinctly convex ventrally; anterolateral apodemes distinct.

Male genitalia. Pygofer 5.8 mm long and 4.2 mm wide, stout, subcordiform, strongly narrowing posteriorly in ventral and dorsal views (Fig. 2D and G); anal styles relatively large, less sclerotised, densely covered with short setae apically (Fig. 2D–G); apical stylus lightly sclerotised, digitiform (Fig. 2G); basal lobes less developed, inconspicuous, obliquely prostrating to side walls of pygofer (Fig. 2D and E); upper lobes absent; distal shoulders elongately tuberculate in ventral view (Fig. 2D) and somewhat protruded and widly rounded at apex in lateral view (Fig. 2F). Uncus with basal part swollen and transversely oval (Fig. 2D); claspers slender, straightly protruding posterolaterally, roundly obtuse at apices (Fig. 2D and E) and slightly bent inwards at sharp apices in lateral view (Fig. 2F). Aedeagus rather thin and slender, gradually tapering apically (Fig. 2I and K); in lateral view, concave at basal third of dorsal surface and distinctly curved ventrally in apical third (Fig. 2J).

Variations. All male types without evident variations, except the holotype faintly tinged fuscous in central part of pronotal disc and posterior part of pronotal collar (all male paratypes without such kind of faint cloud).

**Female** (Fig. 1C and D). Measurements (n = 3) as shown in Table 2. Ratios of different body parts: (pronotal length)/(head length) = 2.6; (mesonotal length excluding cruciform elevation)/(pronotal length) = 1.3; (abdominal length)/(head + pronotal + mesonotal length) = 1.0; (head width)/(pronotal width) = 0.8; (head width)/(mesonotal width) = 1.1; (abdominal tergite III width)/(mesonotal width) = 1.2; (forewing length)/(forewing width) = 3.2.

**Figure 3.**
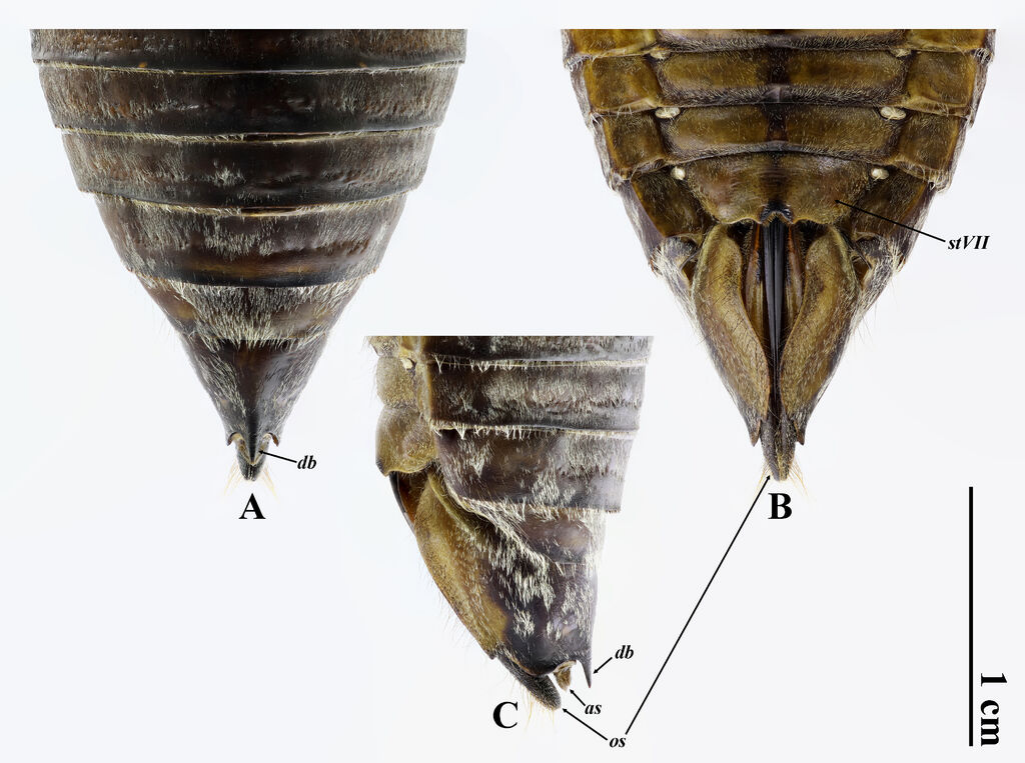
Female terminalia of *Macrosemiafengi* Wang **sp. nov.**, paratype: **A** dorsal view; **B** ventral view; **C** lateral view. Abbreviations: as: anal styles; db: dorsal beak; stVII: sternite VII; os: ovipositor sheath.

Rostrum extending slightly beyond posterior margin of abdominal sternite II; abdomen subconical, strongly converging apically; operculum short, slightly emarginate sublaterally at posterior margin, extending slightly beyond posterior margin of abdominal sternite II and separated from each other a little more than 1.5 times of one width of them; abdominal tergite III 1.2 times as wide as mesonotum; abdominal sternite VII (Fig. 3B) subroundly incised at middle of posterior margin, with paired protuberances flanked incision; abdominal tergite IX with dorsal beak (Fig. 3A and C) somewhat elongate, roundly sharp, almost as long as anal styles; ovipositor sheath (Fig. 3A–C) blackish, extending a little beyond dorsal beak.

#### Diagnosis

*Macrosemiafengi* Wang **sp. nov.** is easily distinguished from other members in this genus by the combination of the following characters: pronotum almost entirely brown, only particularly with paired blackish submedian spots near ambient fissure at pronotal disc (other congeners (except *M.perakana*) have more complex markings on pronotum, at least with a distinct pair of blackish submedian fasciae); forewing with infuscations only present on r and r-m crossveins, while absent on apices of longitudinal veins of apical cells (same as in *M.beaudouini*, *M.divergens*, *M.juno* and *M.tonkiniana*) (*M.anhweiensis* and *M.kiangsuensis* have forewings with infuscations present on r and r-m crossveins, as well as on apices of longitudinal veins of apical cells) (*M.assamensis*, *M.kareisana*, *M.khuanae*, *M.lamdongensis*, *M.matsumurai*, *M.perakana*, *M.sapaensis*, *M.saturata*, *M.suavicolor* and *M.umbrata* have forewings with infuscations present on r, r-m, m and m-cu crossveins, as well as on apices of longitudinal veins of apical cells); uncal claspers slender, straightly protruding posterolaterally, roundly obtuse at apices and slightly bent inwards at sharp apices in lateral view (*M.beaudouini*, *M.divergens*, *M.juno* and *M.tonkiniana* have quite broad uncal claspers).

#### Etymology

The new species is dedicated to Mr. Lei Feng (Weifang, China), a Chinese amateur obsessing with cicadas, for his help to my taxonomic study on Cicadidae. The name is a noun in the genitive case. “冯氏大马蝉” is proposed for the Chinese common name of this new species.

#### Distribution

**Figure 4.**
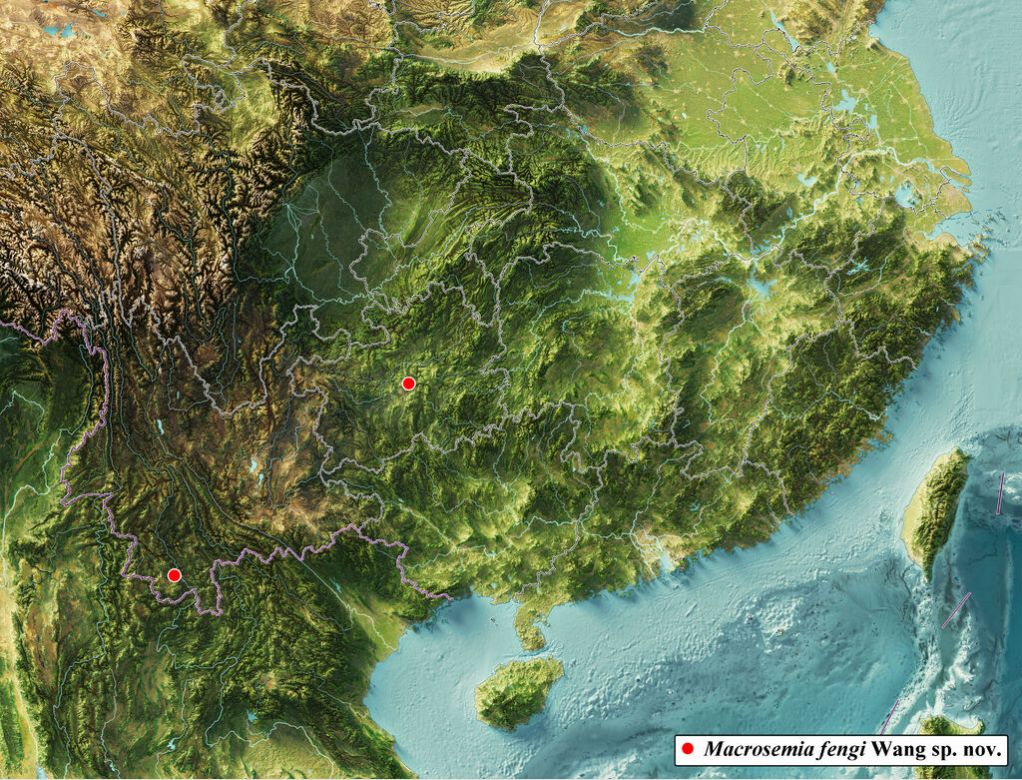
Distribution map of *Macrosemiafengi* Wang **sp. nov.**

China (Yunnan, Guizhou) (Fig. 4).

## Supplementary Material

XML Treatment for Macrosemia fengi

## Figures and Tables

**Table 1.** Morphometric values (mm) for each male type of *Macrosemiafengi* Wang **sp. nov.**

**Specimen ID**	WCBA00170 (♂, HT)	WCBA00171 (♂, PT)	WCBA00173 (♂, PT)	WCBA00175 (♂, PT)	**Average**
**body length**	43.2	46.5	42.5	45.3	44.4
**head length**	4.0	4.5	4.0	4.4	4.2
**pronotal length**	7.8	8.6	7.7	8.3	8.1
**mesonotal length**	11.0	12.2	10.8	11.6	11.4
**forewing length**	55.7	60.9	54.9	60.1	57.9
**abdominal length**	20.4	21.2	20.0	21.0	20.7
**head width**	15.8	16.2	15.5	16.1	15.9
**pronotal width**	17.2	17.8	17.0	17.6	17.4
**mesonotal width**	14.9	15.7	14.5	15.5	15.2
**forewing width**	16.0	18.9	15.1	18.5	17.1
**abdominal tergite III width**	16.2	16.6	16.0	16.5	16.3

**Table 2.** Morphometric values (mm) for each female type of *Macrosemiafengi* Wang **sp. nov.**

**Specimen ID**	WCBA00172 (♀, PT)	WCBA00174 (♀, PT)	WCBA00176 (♀, PT)	**Average**
**body length**	47.8	45.4	47	46.7
**head length**	3.3	2.8	3.1	3.1
**pronotal length**	8.3	7.8	8.2	8.1
**mesonotal length**	12.7	12.2	12.6	12.5
**forewing length**	63.4	60.6	62.1	62.0
**abdominal length**	23.5	22.6	23.1	23.1
**head width**	16.2	15.6	15.9	15.9
**pronotal width**	19.0	18.4	18.8	18.7
**mesonotal width**	14.7	14.2	14.3	14.4
**forewing width**	20.1	18.7	19.7	19.5
**abdominal tergite III width**	17.8	17.3	17.6	17.6
